# Gas6 is dispensable for pubertal mammary gland development

**DOI:** 10.1371/journal.pone.0208550

**Published:** 2018-12-11

**Authors:** Kylie L. Mills, Angelica M. Gomes, Courtney R. Standlee, Michelle D. Rojo, Peter Carmeliet, Zhen Lin, Heather L. Machado

**Affiliations:** 1 Department of Biochemistry and Molecular Biology, Tulane Cancer Center, Tulane School of Medicine, New Orleans, LA, United States of America; 2 Laboratory of Angiogenesis and Vascular Metabolism,VIB-KU Leuven Center for Cancer Biology, Leuven, Belgium; 3 State Key Laboratory of Ophthalmology, Zhongsan Ophthalmic Center, Sun Yat-Sen University, Guangzhou, China; 4 Laboratory of Angiogenesis and Vascular Metabolism, Department of Oncology and Leuven Cancer Institute (LKI), KU Leuven, Leuven, Belgium; 5 Department of Pathology, Tulane Cancer Center, Tulane School of Medicine, New Orleans, LA, United States of America; University of Tennessee Health Science Center, UNITED STATES

## Abstract

Mammary gland development is a complex and dynamic process that occurs mainly postnatally. Ductal elongation and branching morphogenesis are regulated by a plethora of factors, including cytokines, hormones, growth factors and the extracellular matrix. Gas6 is a secreted gamma-carboxylated protein that binds to a family of receptors tyrosine kinase receptors known as the TAMR family (Tyro3, Axl, Mer). Gas6 function in developmental processes has been shown in nervous, reproductive and immune systems. In this study, we found that Gas6 is highly expressed in virgin adult mammary glands but declines during pregnancy and lactation. Specifically, Gas6 is highly expressed in luminal and basal mammary epithelial cells during puberty and adulthood, while TAMR expression is low. Mammary whole mount analysis revealed that Gas6 germline deletion does not impact ductal elongation, branching morphogenesis or terminal end bud formation. Masson’s trichrome staining showed that collagen deposition is similar in Gas6^-/-^ mice as compared to wildtype mice. Gas6^-/-^ mammary glands presented an organized luminal and myoepithelial bilayer of cells, and the proportion of mammary stem cells was unchanged in Gas6^-/-^ mammary glands as compared to wildtype. Finally, proliferation of epithelial cells and macrophage number were similar in both groups. These studies suggest that Gas6 is not essential for pubertal mammary gland development in nulliparous mice.

## Introduction

Mammary gland development is a carefully orchestrated process that occurs primarily postnatally. This developmental program is characterized by ductal elongation and branching morphogenesis that results in a functional epithelial ductal tree. Several factors coordinate the invasion of epithelial cells through the mammary fat pad including local growth factors and cytokines, circulating hormones, extracellular matrix proteins and epithelial-stroma cell interactions. During puberty, systemic hormones such as estrogen drive the formation of highly proliferative structures at the tips of the epithelial ducts called terminal end buds (TEBs). Epithelial cells of TEBs are also highly invasive and promote the extracellular matrix remodeling necessary for ductal elongation and branching. Additionally, TEBs cells undergo a regulated process of apoptosis in order to give rise to lumen formation [1–3]. After puberty, the mammary ducts reach the end of the fat pad and TEB regression occurs. As a result, a mature ductal tree, mostly quiescent until pregnancy, is formed. The adult mammary gland is comprised of a bilayer of cells with unique characteristics. The luminal cell layer that lines the ducts contain hormone receptor-positive cells that express cytokeratin (CK) 8 and CK18 while the basal layer is in contact with the basement membrane and consists of myoepithelial cells that express CK5 and CK14, as well as stem cells.

Growth arrest-specific 6 (Gas6) is a vitamin K-dependent cytokine that binds to a family of type I transmembrane receptor tyrosine kinases comprised of Tyro3, Axl, and Mer (TAMR family). Protein S (Pros1), which primarily functions as an anti-coagulant, is also a ligand for TAMRs. While Gas6 has the highest affinity for Axl, Pros1 preferentially binds to Tyro3 and Mer [4–7]. Gas6 was first identified in 1988 as an upregulated gene in growth-arrested fibroblasts [8]. Since then, numerous studies have described Gas6 function in diverse physiological and pathological processes [5, 9, 10]. Gas6 signaling has been show to modulate proliferation, apoptosis, invasion, efferocytosis, and migration [11–13]. Perhaps the most well known function of Gas6/TAMR signaling is the regulation of innate immune responses in several cell types. Additionally, Gas6/Axl signaling has been shown to be associated with poor prognosis of several cancer types [14–16]. Interestingly, in breast cancer patients, Gas6 mRNA expression correlates with progesterone receptor B and with good prognostic factors [17], whereas Axl expression has been correlated with poor overall survival [15].

The function of the TAMR family and its ligands in virgin mammary gland development is not well understood. Sandhal *et al* demonstrated that Mer is essential for the clearance of apoptotic cells in the post-lactational mammary gland, while dual deletion of Axl and Tyro3 had no effect. Additionally, they showed that during puberty, Mer is not required for branching morphogenesis or growth of mammary epithelium [18]. Goyette *et al* showed that adult virgin mammary glands lacking Gas6 appeared normal [15], however Gas6 function in ductal elongation and branching morphogenesis during pubertal mammary gland development has not been well-studied.

In this study, we investigate whether Gas6 signaling modulates pubertal mouse mammary gland development in nulliparous mice. As Gas6 signaling is a crucial mediator of proliferation and apoptosis, we hypothesized that this pathway may regulate ductal morphogenesis, which is tightly regulated by a balance of proliferation and apoptosis. While we observed that Gas6 was highly expressed in both luminal and basal cells of the mammary gland, TAMR expression was low. We showed that deletion of Gas6 does not alter ductal elongation or branching morphogenesis, and we characterized ductal morphology, collagen deposition, macrophage number, proliferation and basal and luminal cell organization.

## Results

### Gas6 is highly expressed in basal and luminal cells during mammary gland development

**Fig 1:**
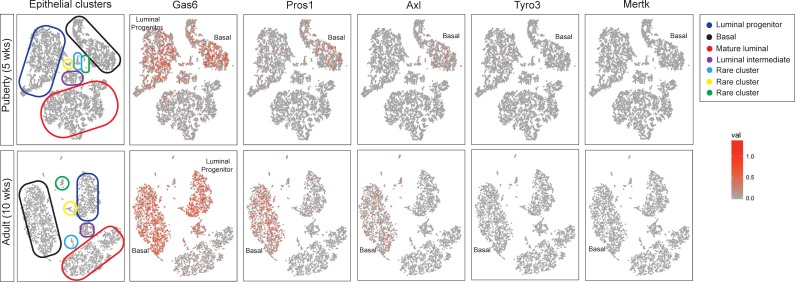
TAMR expression and their ligands in mammary epithelial cells during mammary gland development. 2-D t-SNE projection plots of scRNA-seq data from mammary epithelial cells isolated from pubertal and adult mouse mammary gland. *Tyro3*, *Axl*, *Mertk*, *Gas6* and *Pros1* expression in different epithelial cell clusters [20]. Each cell was colored by normalized expression of the genes in the cell. Color scale represents the normalized UMI (unique molecular identifier) counts for each gene under a log10 scale.

**Fig 2:**
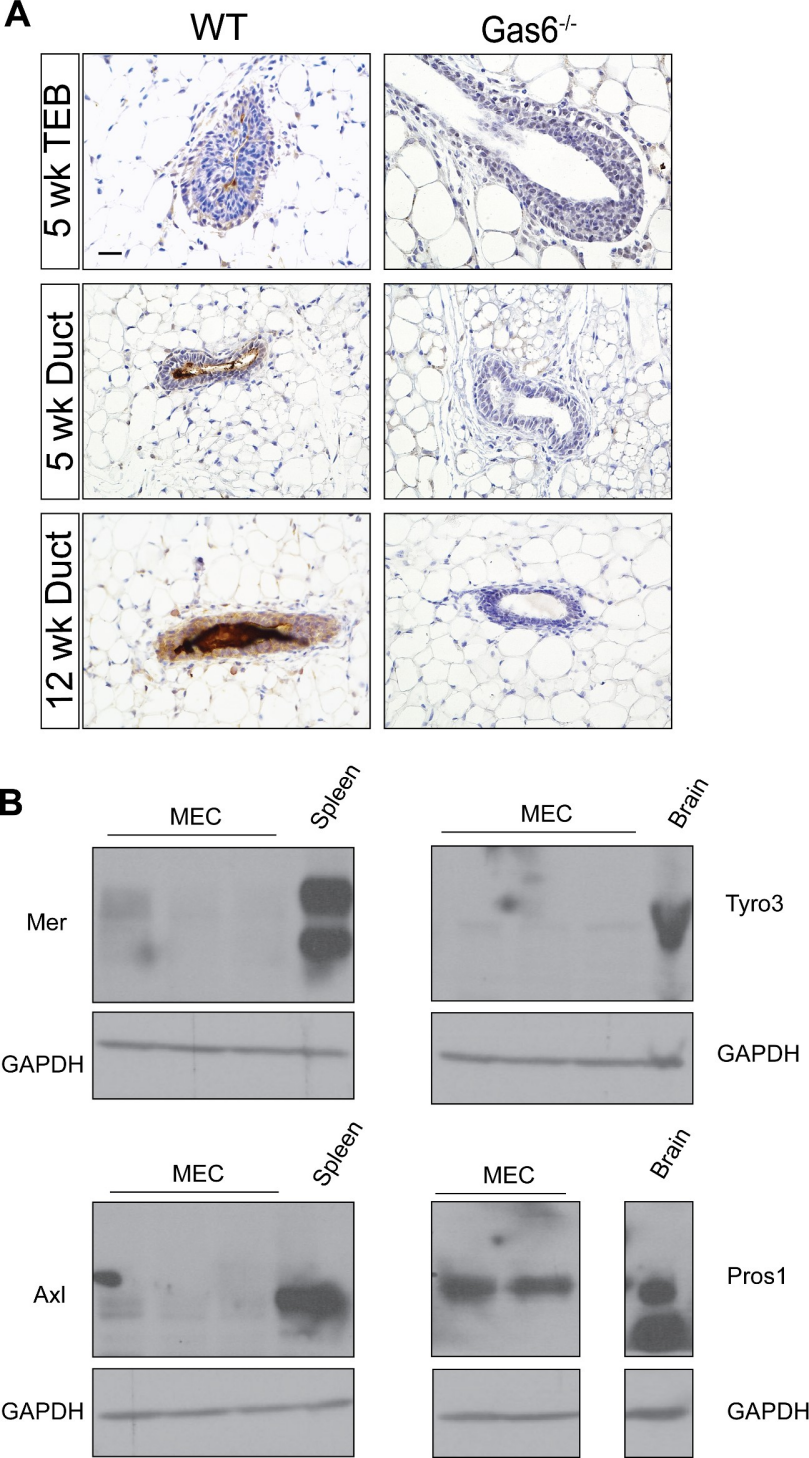
TAMR protein expression and their ligands in normal mammary gland. (A) Mammary glands (n = 3 per time point) were harvested in diestrus from 5 and 12 week old animals and stained with an antibody to Gas6. Representative images of mouse mammary tissue showing Gas6 expression in mammary epithelial cells and mammary ductal lumen. Scale bar = 20 μm. (B) Western blot analysis of TAMR (n = 3) and Pros1 (n = 2) in epithelial organoids isolated from adult animals. Spleen and brain were used as positive controls. Uncropped blots are depicted in S2 Fig.

To initiate these studies, we analyzed Gas6 gene expression in adult mammary glands from virgin, pregnant, and lactating mice from publically available transcriptomic data [19]. Gas6 is highly expressed in virgin adult glands (10 and 12 weeks), however its expression declines during pregnancy and lactation (S1 Fig). Therefore, the remainder of this study is focused on understanding Gas6 function during mammary gland morphogenesis in pubertal and nulliparous adult mice. We next asked whether Gas6 and the TAMR family are expressed in heterogeneous epithelial cell populations during puberty and adult mammary glands by analyzing recent single cell RNA sequencing (scRNA-seq) data [20]. We analyzed these data by using the 10X Genomics cell ranger software and explored target gene expression at the single cell level. Cells were first clustered using t-SNE projection and then colored by normalized gene expression values. Among all the genes analyzed, *Gas6* was the most abundant, being present in luminal progenitor, luminal intermediate, mature luminal and basal cells, as well as previously identified rare clusters in pubertal and adult mammary glands. Interestingly, few mammary epithelial cells expressed the TAMRs, with *Axl* being the most abundant receptor (Fig 1). *Axl* was primarily expressed in the basal population in pubertal and adult mammary glands. Notably, *Pros1* expression was observed mainly in basal cells. We also analyzed Gas6 protein expression in pubertal (5 weeks) and adult (12 weeks) mouse mammary glands by immunohistochemistry. In pubertal mice, there was intense Gas6 staining in the lumens of the mammary ducts and TEBs, while staining was relatively low in the epithelial cells (Fig 2A). These data suggest that Gas6 is rapidly secreted during ductal elongation. Gas6 staining was strongest in adult mammary glands in the myoepithelial cells, luminal cells, and the lumens, consistent with the scRNA-seq data. Staining of mammary glands from Gas6^-/-^ mice confirmed the specificity of the antibody (Fig 2A). To investigate TAMR and Pros1 protein expression, we isolated mammary epithelial organoids from adult wildtype mice and performed western blotting. In support of the scRNA-seq data, low levels of Mer, Axl and Tyro3 were expressed in mammary epithelial cells, as compared to spleen or brain. Similar to Gas6, Pros1 protein expression was high in mammary epithelial cells (Fig 2B).

### Gas6 deletion does not alter ductal morphogenesis

**Fig 3:**
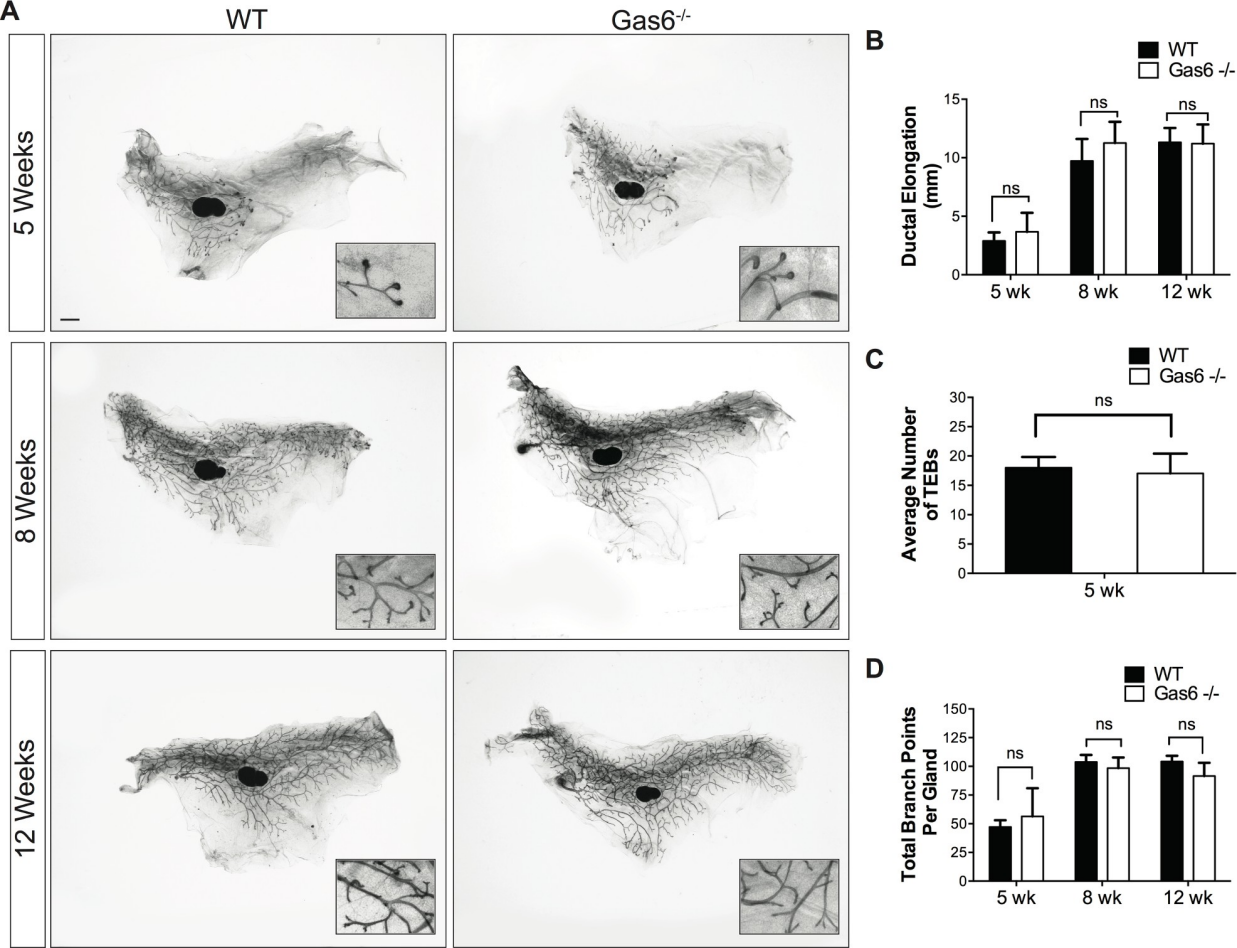
Ductal morphogenesis is normal in Gas6^-/-^ mammary glands. Mammary glands (5–7 mice per timepoint, 2 mammary glands per mouse) were harvested in diestrus from 5, 8 and 12 week old animals. (A) Carmine alum stained whole mounts of mammary glands from wildtype and Gas6^-/-^ animals. Scale bar = 1 mm. (B) Ductal elongation was quantified by measuring the distance from lymph node to the most distal tip of a duct or TEB. (5 wk p = 0.08; 8 wk p = 0.06; 12 wk p = 0.86). (C) Quantification of TEB number in wildtype and Gas6^-/-^ glands (p = 0.49). (D) Branching morphogenesis was quantified by counting the branch points in the entire gland (5 wk p = 0.48; 8 wk p = 0.48; 12 wk p = 0.11).

To understand the function of Gas6 in mammopoiesis, we evaluated *Gas6* germline knockout mice for defects in mammary gland formation. Mammary glands were harvested from 5, 8 and 12 week old diestrus-staged virgin female mice. Ductal elongation was determined by measuring the distance from the center of the lymph node to the most distal tip of a duct or TEB. As shown in Fig 3, there was no significant difference in ductal elongation in Gas6^-/-^ mammary glands as compared to wildtype (Fig 3A and 3B). The number of TEBs in Gas6^-/-^ glands were comparable to those in wildtype, and TEB morphology appeared normal (Fig 3B and 3C). Finally, we analyzed branching morphogenesis by counting branch points in the entire mammary gland. Quantification of branch points showed that there was no significant difference in branching morphogenesis in wildtype and Gas6^-/-^ mice at all stages of ductal morphogenesis (Fig 3D). These data were verified in Gas6^-/-^ mice that were backcrossed to the Balb/c genetic strain. In Balb/c mice, there were no observed changes in ductal elongation or TEB formation in pubertal (S3 Fig) mammary glands. Collectively, these results suggest that Gas6 is not required for mammary ductal outgrowth or branching morphogenesis.

**Fig 4:**
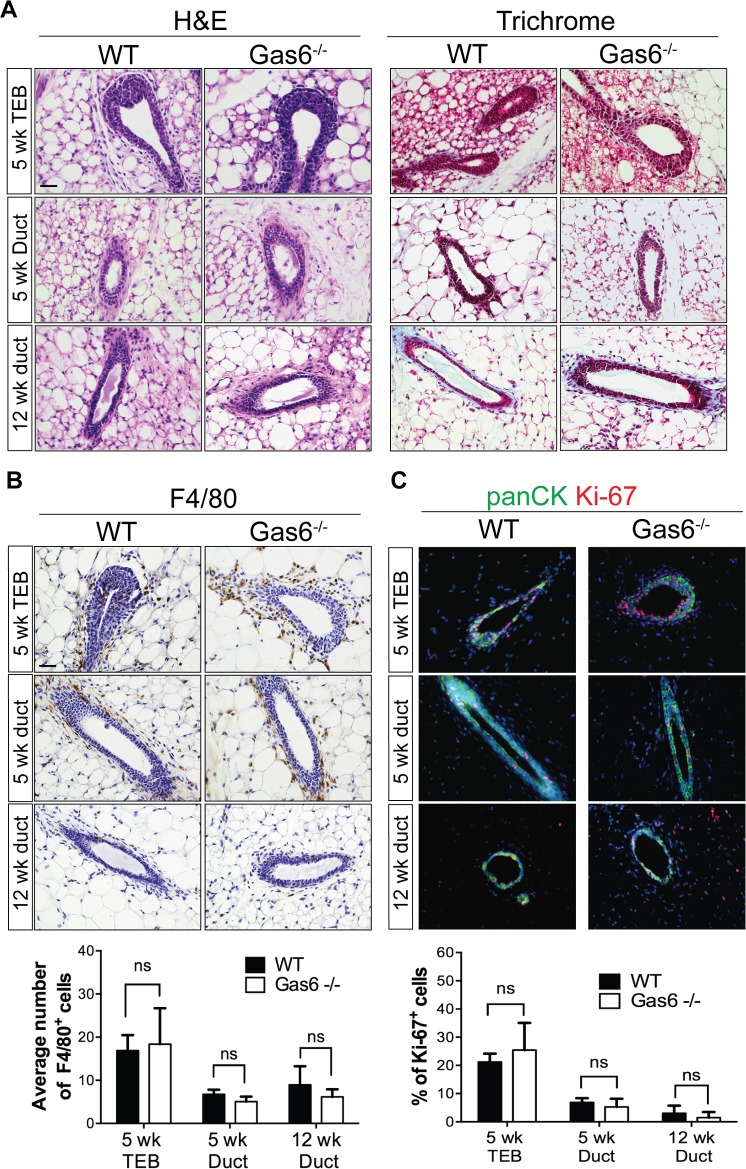
Histological characterization of Gas6^-/-^ mammary ducts. (A) Representative images of mouse mammary tissue stained with H&E and Masson’s trichrome (3 glands from 3 mice). Scale bar = 20 μm. (B) Representative images of mouse mammary tissue from wildtype and Gas6^-/-^ animals stained with an antibody for F4/80. Graph depicts quantification of the average number of F4/80^+^ cells in wild type and Gas6^-/-^ mammary sections. For each gland, a minimum of 4 ducts and 4 TEBs were counted from 3 glands (3 mice) for each group (5 wk TEB p = 0.81; 5 wk duct p = 0.25; 12 wk duct p = 0.56). Scale bar = 20 μm. (C) Representative immunofluorescence images of mammary sections stained with antibodies for Ki-67 (red) and panCK (green) are depicted. Graph shows quantification of the average number of Ki-67^+^ cells in WT (5 week, n = 7; 12 week, n = 5) and Gas6^-/-^ (5 week, n = 5; 12 week, n = 4) mammary glands, where *n* is the number of animals. For each gland, a minimum of 4 ducts and 4 TEBs were counted per animal (5 week TEB p = 0.41; 5 week duct p = 0.46; 12 week duct p = 0.64). Scale bar = 20 μm.

To further characterize the Gas6^-/-^ mice, we histologically examined the mammary glands at 5 and 12 weeks of age. Hematoxylin and eosin staining showed no morphological changes in Gas6^-/-^ mammary glands as compared to wildtype (Fig 4A). Alterations in the stroma were analyzed by staining with Masson’s trichrome and showed similar collagen deposition in Gas6^-/-^ mammary glands as compared to wildtype (Fig 4A). Since Gas6 is a well-known regulator of innate immune responses, we analyzed macrophage content by F4/80 staining. Quantification of F4/80^+^ cells revealed similar numbers of macrophages surrounding the ducts or TEBs in wildtype and Gas6^-/-^ mice in the time points analyzed (Fig 4B). Given that Gas6 is a well-known mitogenic factor for several cell types [11, 21], we stained mammary glands with an antibody to detect proliferation (Ki-67). Fig 4 shows that proliferation in mammary epithelial cells from wildtype and Gas6^-/-^ is similar during puberty and adulthood (Fig 4C). Together, these results indicate that Gas6 is not required for mammary gland morphogenesis.

**Fig 5:**
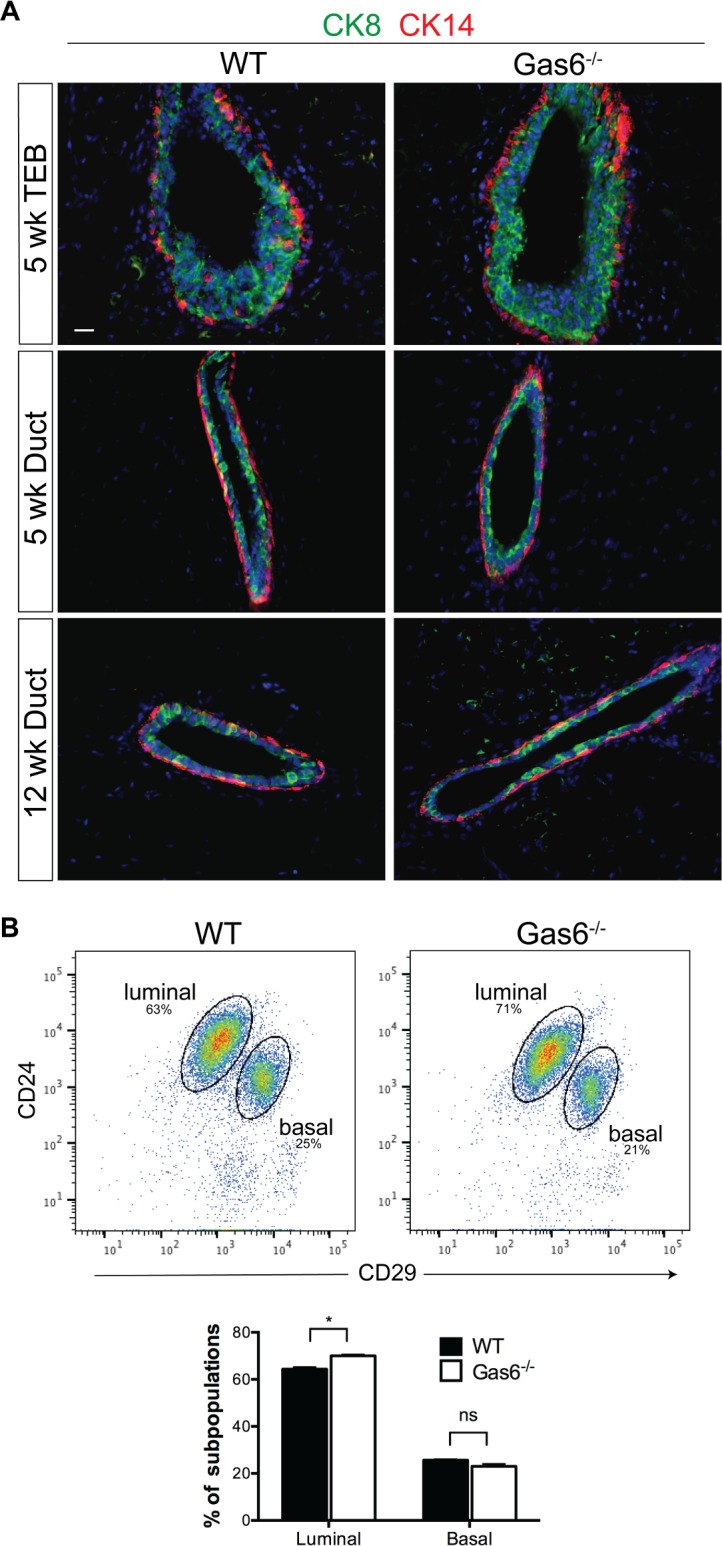
Gas6^-/-^ mammary glands are comprised of normal luminal and basal cell layers. (A) Representative images of mammary glands from wild type and Gas6^-/-^ mice stained with antibodies to CK14 (red) and CK8 (green) (3 glands from 3 mice). Scale bar = 20 μm. (n = 3) (B) Dot plots depict the percentages of LIN^−^CD24^hi^CD29^lo^ (luminal) or LIN^−^CD24^+^CD29^hi^ (basal) cell population in mammary epithelial cells from wild type and Gas6^-/-^ animals (n = 3). Graph depicts the percentages of luminal and basal cells (mean and SEM) from wildtype and Gas6^-/-^ mammary glands from 3 independent experiments (*p< 0.05).

The mammary ducts are comprised of a bilayer of basal and luminal cells, which contain mammary stem cells (MaSCs), progenitor cells and differentiated progeny. To determine whether Gas6 regulates the organization of luminal and basal epithelial layers, we stained mammary glands with antibodies that detect myoepithelial (CK14) and luminal cells (CK8). Immunostaining showed an intact and organized CK14^+^ myoepithelial cell layer surrounding the CK8^+^ luminal cells in wildtype and Gas6^-/-^ mice (Fig 5A), suggesting that Gas6 is not required for structural organization of the bilayered mammary duct. Luminal and basal cells can also be distinguished by flow cytometry, in which CD24^+^CD29^lo^ cells represent the luminal cell lineage, and CD24^hi^CD29^hi^ cells comprise the basal cell lineage and include MaSCs [22]. Analysis of these populations in Gas6^-/-^ glands showed that while the proportion of luminal cells increased (64% to 70%), there was no significant difference in basal cells (including MaSCs) in wildtype and Gas6^-/-^ mammary glands (Fig 5B). These results suggest that lineage composition is not altered in Gas6-deleted mammary glands.

## Discussion

The utilization of genetic mouse models has significantly advanced our understanding of the molecular drivers of mammary gland development. In this study, we characterized the phenotype of germline deletion of Gas6 during different stages of ductal morphogenesis. These studies were performed with scientific rigor analyzing a sufficient number of animals in two different genetic strains. Gas6 was highly expressed in basal and luminal epithelial cells during development, suggesting it may regulate mammopoiesis. Interestingly, Gas6 was rapidly secreted by the epithelial cells resulting in the accumulation of Gas6 in the ductal lumens (Fig 2). Despite abundant expression and secretion, genetic ablation of Gas6 did not alter ductal elongation, branching morphogenesis, or cell proliferation (Figs 3–5).

Receptor tyrosine kinases are crucial regulators of mammary function [23–25]. Early studies by Chodosh *et al* used northern blotting to analyze various RTKs at different stages of development, including *Axl* and *Tyro3*. While *Axl* was detected during puberty and adulthood, its expression decreased during pregnancy. In contrast, *Tyro3* was highly expressed during pregnancy [26]. These results suggest that Gas6 may activate different receptors during distinct stages of mammary gland development. Analysis of publically available scRNA-seq data identified TAMR expression in several epithelial cell clusters present in pubertal and adult mammary glands. In support of previous studies, *Axl* was detected in pubertal and adult mammary epithelial cells while *Tyro3* and *Mertk* expression was rare. Interestingly, *Axl* expression was restricted to a subpopulation of basal cells (Fig 1). Since MaSCs with self-renewing properties and *in vivo* repopulating activity are also expressed in a subpopulation of basal cells, it is tempting to speculate that Axl may be a putative stem cell marker. Indeed, Axl has been suggested to be a cancer stem cell marker [27, 28], however it is unclear as to whether Axl is a true MaSC marker in the normal mammary gland.

Despite the fact that Gas6 is highly expressed in mammary epithelial cells during development, Gas6 loss did not alter ductal morphogenesis. In agreement with our findings, Goyette *et al* recently showed that mammary gland outgrowth in adult mammary glands lacking Axl or Gas6 appeared normal, although ductal elongation and branching morphogenesis in estrus-staged animals was not examined [15]. Also consistent with our findings, they showed that there was no change in proliferation in mammary intraepithelial neoplasias of MMTV-Neu;Gas6^-/-^ mice [15]. Nonetheless, the accumulation of secreted Gas6 in the mammary ducts, and abundant expression in basal cells, luminal progenitors, and mature luminal cells suggest that Gas6 contributes to mammary function in some context. It is possible that Gas6 germline deletion may trigger alternative compensatory mechanisms that overcome the loss of Gas6 in early pubertal development. It would be interesting to use an inducible system to evaluate the consequences of loss of Gas6 at specific stages of development. Although Gas6 was historically believed to be the only ligand for Axl, recent studies suggest that Pros1 can also bind and activate Axl [29]. In glioblastoma, macrophage-derived Pros1 induced Axl phosphorylation and consequent tumor growth in an NFκB-dependent manner [29]. In the mammary gland, Pros1 expression paralleled Axl expression and was primarily expressed in basal cells (Fig 1). While it remains unclear as to whether Gas6 or Pros1 interact with Axl in the mammary gland, it is possible that Pros1 can compensate for the loss of Gas6 in Gas6^-/-^ mice by activating Axl, and thus masking potential mammary gland defects in Gas6-null mammary glands. Notably, we did not detect an increase in Pros1 expression in adult Gas6^-/-^ mammary glands as compared to wildtype (S2 Fig). Additional studies using genetic approaches will be crucial to dissect these mechanisms. In conclusion, we report that despite abundant expression in pubertal and adult mammary glands, Gas6 is dispensable for pubertal mammary gland development.

## Materials and methods

### Animal models

Mice were housed in a pathogen-free facility as recommended by the NIH Guide for the Care and Use of Experimental Animals. All animal care and procedures were approved by the Tulane School of Medicine Institutional Animal Care and Use Committee (Protocol #4309R). *Gas6*^*-/-*^ mice (C57BL/6J) were maintained and genotyped as previously described [30]. Additionally, *Gas6*^*-/-*^ mice were backcrossed eight generations to the BALB/cAnHsd strain (Envigo) and genotyped for all experiments. All experiments were performed in accordance with relevant guidelines and regulations.

### Single cell RNA-seq data analysis

The single cell RNA sequencing data of isolated epithelial cells from the mammary glands of FvB/NJ mice in puberty and adult stages were obtained through the National Center for Biotechnology Information (GSE103275). The gene expression values were generated by the 10X Genomics Chromium Cell Ranger pipeline and imported into the R software with the Cell Ranger R kit. High-dimensional gene expression profiles were reduced to a two-dimensional representation using the t-SNE (t-Distributed Stochastic Neighbor Embedding) projection. Cells were then colored by normalized expression of the genes in the cell.

### Whole mounts analysis

For whole mount analysis, inguinal (#4) mammary glands were harvested from 5, 8 and 12-week virgin female mice in diestrus (5–7 mice per timepoint, 2 mammary glands per mouse). The glands were fixed in cold 4% paraformaldehyde for 2 hours on ice, and stained with Carmine alum overnight. Glands were dehydrated using 70%, 95% and 100% ethanol and cleared in xylene. Mammary glands were mounted with permount (Fischer Scientific, SP15) and imaged using a Leica M165FC stereoscope. Ductal elongation was quantified with NIS-Elements Basic Research Software (Nikon Instruments) by measuring the distance from the center of the lymph node to the last end bud or terminal ductal unit. Branching morphogenesis was quantified by counting all branch points in the entire mammary gland. Mammary glands were subsequently paraffin embedded for histological analysis. Analyses were performed on Gas6^-/-^ and wildtype mice in the C57BL/6 strain, and data were confirmed in Gas6^-/-^ and wildtype Balb/c mice (S3 Fig).

### Histological analysis and immunostaining

Paraffin embedded mammary glands (3 mice per group) were sectioned at 5 μm, deparaffinized, rehydrated and stained with hematoxylin and eosin. Masson’s trichrome staining was performed according to the manufacturer’s instructions (Sigma HT15). For detection of Gas6, antigen retrieval was performed in the microwave using 10 mM sodium citrate for 20 minutes. F4/80 staining was performed without antigen retrieval as previously described [31]. Peroxidases were quenched with 3% H_2_O_2_ in methanol, blocked with 5% BSA in PBS containing 0.05% Tween (Gas6) or M.O.M. blocking reagent (F4/80; Vector Laboratories), and incubated with antibodies overnight at 4°C. Antibodies and dilutions are listed in S1 Table. The next day, slides were washed with PBS and incubated with a biotinylated antibody (1:500) (Vector Laboratories) for 30 min. Slides were washed with PBS, incubated for 10 min with the VECTASTAIN Elite ABC-HRP reagent, R.T.U (Vector Laboratories), and developed using a DAB peroxidase substrate kit (Vector Laboratories). Glands were counterstained with hematoxylin, dehydrated and mounted with permount (Fisher Scientific). For quantification of macrophages, 4 ducts and 4 TEBs from each gland were counted (3 mice). For immunofluorescence, tissues were stained with antibodies to CK14, CK8, pan-CK and Ki-67 as previously described [31] (S1 Table). The next day, slides were washed with PBS and stained with Alexa Fluor-conjugated secondary antibodies (diluted 1:500; Thermo Fisher Scientific) for 2 hours at room temperature. Slides were mounted with Prolong Diamond Antifade Mountant containing DAPI (Thermo Fisher). Images were acquired using a Nikon Eclipse microscope (Nikon Instruments) under a 40X objective. For quantification of proliferation, the number of Ki-67^+^ cells was calculated as a percentage of total epithelial cells. Pan-CK staining was used to identify the epithelium.

### Mammary epithelial cell isolation

Mammary glands (#3, #4 and #5 without lymph nodes) were collected from adult virgin females (10–14 weeks old), minced using a sterile razor blade, and digested for 1 hour with shaking at 37°C in DMEM/F12 containing 2 mg/ml of collagenase A (Roche Diagnostics #10050021) and 1X antibiotic-antimycotic. Cells were washed with DMEM/F12 containing 5% FBS, centrifuged at 600 x g for 10 minutes, and single epithelial cells were purified as previously described [32]. Epithelial organoids were used for western blot and mammary epithelial cells were used for flow cytometer analysis.

### Western blot

Epithelial organoids were lysed in RIPA buffer containing protease and phosphatase inhibitors. Lysates were centrifuged at 14,000 x g at 4°C for 10 minutes. Protein concentration was quantified by using the Pierce BCA Protein Assay Kit according to the manufacturer’s instructions. Equal amounts of protein (60 μg) were separated by a gradient (4–12%) SDS/PAGE. Proteins were transferred to a polyvinylidene difluoride membrane (Biorad) for 1h at 100V. Membranes were blocked with 5% skim milk in TBST. Specific proteins were detected by incubating membranes overnight with antibodies listed in S1 Table. Tyro3, Axl and Mer antibodies were previously characterized [33, 34]. Then, membranes were washed and incubated with HRP-conjugated secondary antibodies (dilution 1:5000) for 1 hour. Bands were detected using an ECL Western Blotting Substrate and CL-XPosure Film.

### Flow cytometry

Single cells were isolated from Gas6^-/-^ or wildtype mice (3 mice per group) and resuspended in HBSS containing 2% FBS and 100 mM Hepes (HBSS^+^) at a concentration of 1 × 10^7^ cells/ml. Antibody staining was performed as previously described [35], and antibodies are listed in S1 Table. Lineage exclusion was achieved with the EasySep Mouse Epithelial Cell Enrichment Kit (Stem Cell Technologies) prior to antibody staining. Cells were filtered with a 35 μm cell strainer, stained with 5 nM SYTOX (Life Technologies), and analyzed by flow cytometry using BD LSRII flow cytometer. Data were analyzed using FlowJo version 10.

### Statistical analysis

Unpaired *t* tests using GraphPad Prism6 were performed for all statistical analyses.

## Supporting information

S1 Fig Graph depicts the relative expression (average + SEM) of *Gas6* in mammary glands from virgin 10 week (V10), virgin 12 week (V12), pregnant day 1 (P1), pregnant day 2 (P2), pregnant day 3 (P3), pregnant day 8.5 (P8.5), pregnant day 12 (P12), pregnant day 14 (P14), pregnant day 17 (P17), lactating day 1 (L1), lactating day 3 (L3), and lactating day 7 (L7). Data were obtained by Stein *et al* using the Affymetrix MG-U74Av2 chip, and normalized signal data was obtained from the original publication [19].
(TIFF)

S2 Fig Western blot analysis of TAMR (n = 3) and Pros1 (n = 2) in epithelial organoids isolated from adult wildtype animals, or Gas6^-/-^ mice (Pros1 only). Images depict a wider view of blots and molecular size markers prior to cropping. GAPDH blot was exposed for 1 second on film while the TAMR and Pros1 blots were exposed for 5 minutes on film. Prior to incubation with primary antibodies, membranes were first cut horizontally so that GAPDH could be detected on the same blot according to its molecular weight. Blots were then cut vertically in order to detect Mer, Axl, Tyro3 and Pros1.
(TIF)

S3 Fig Carmine-stained whole-mount images of the (#4) inguinal mammary glands from WT and Gas6^-/-^ animals (Balb/c strain) at 5 weeks. Mammary glands were harvested from virgin female mice in diestrus. Scale bar = 1 mm.
(EPS)

S1 Table List of antibodies and experimental conditions. (DOCX)
