# Supplementary figures and images for: Gas6 is dispensable for pubertal mammary gland development
Source: PLoS One. 2018 Dec 11;13(12):e0208550. doi: 10.1371/journal.pone.0208550 (PMC6289431; doi:10.1371/journal.pone.0208550)

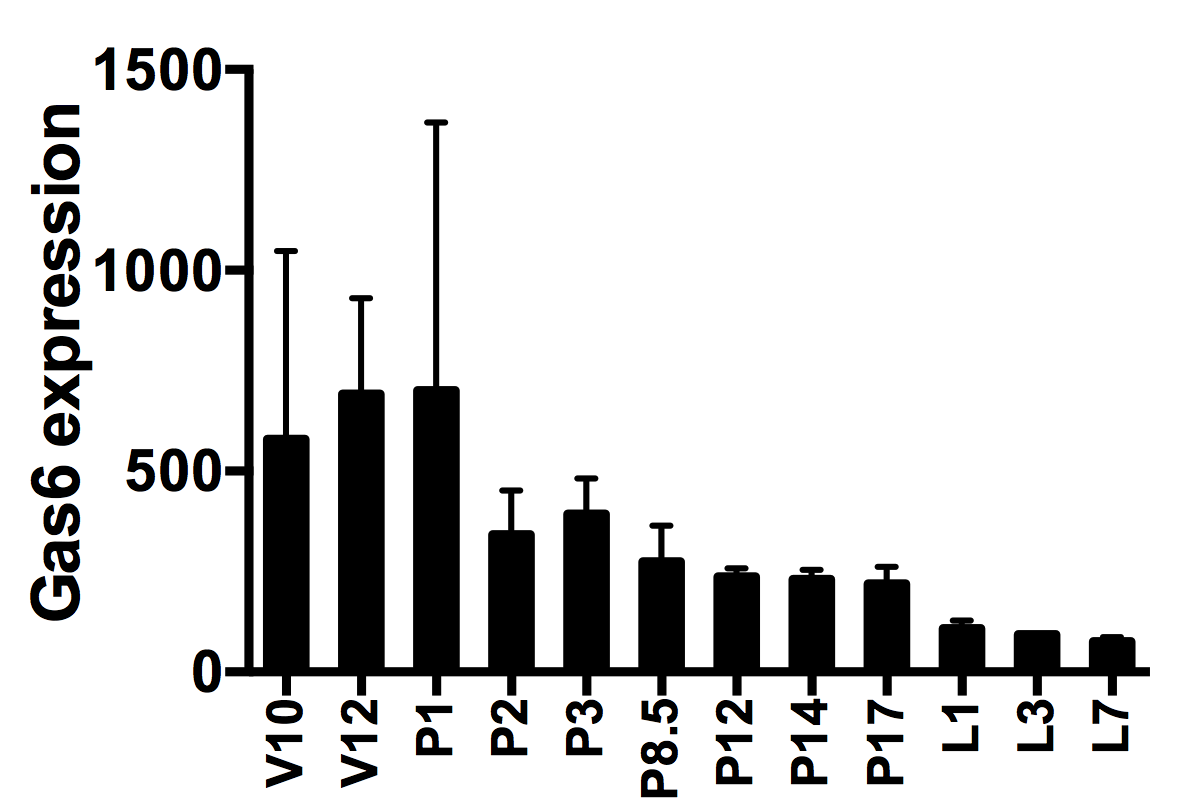

Supplement: S1 Fig — Data were obtained by Stein et al using the Affymetrix MG-U74Av2 chip, and normalized signal data was obtained from the original publication [19]. (TIFF) [file pone.0208550.s001.tiff]

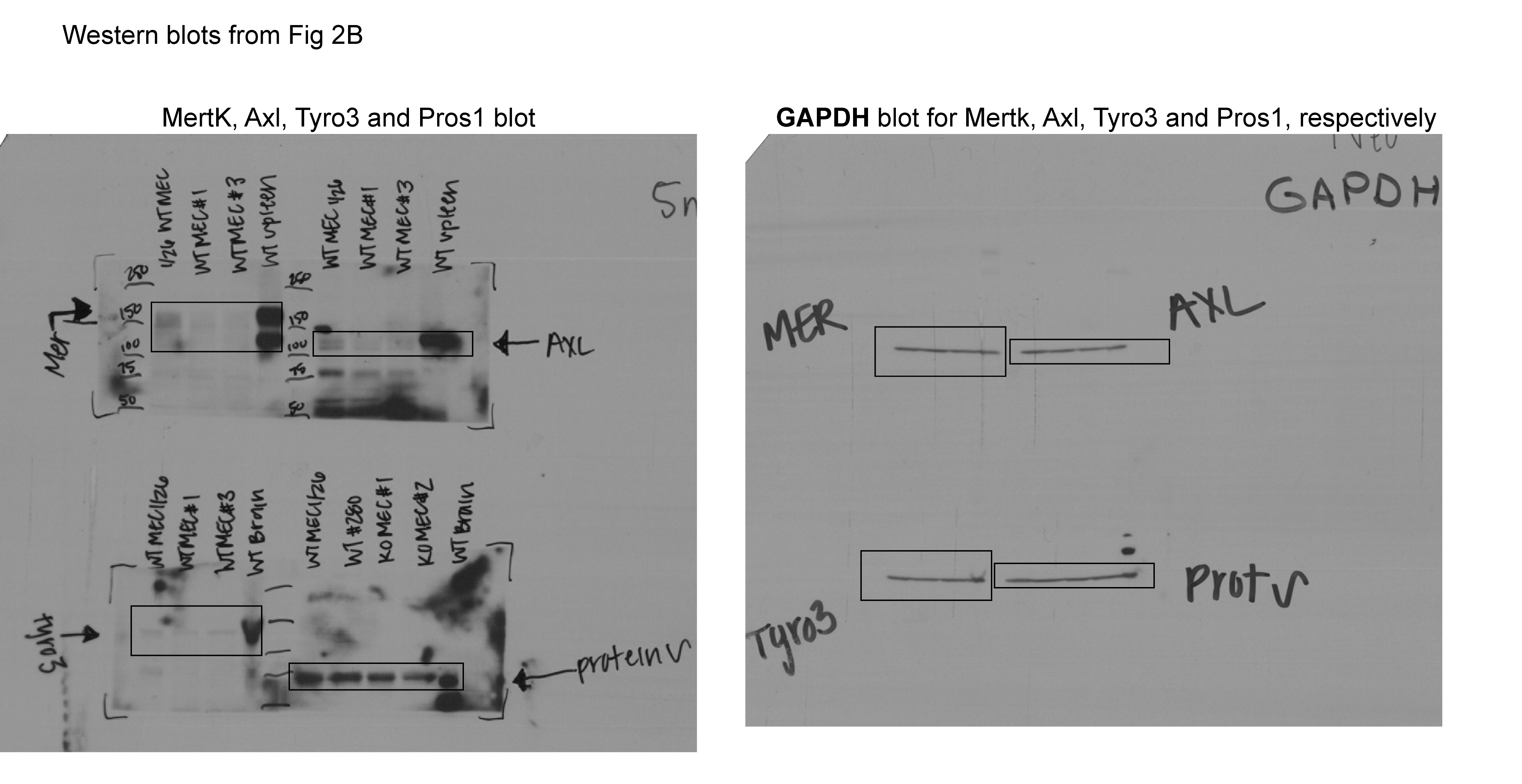

Supplement: S2 Fig — Images depict a wider view of blots and molecular size markers prior to cropping. GAPDH blot was exposed for 1 second on film while the TAMR and Pros1 blots were exposed for 5 minutes on film. Prior to incubation with primary antibodies, membranes were first cut horizontally so that GAPDH could be detected on the same blot according to its molecular weight. Blots were then cut vertically in order to detect Mer, Axl, Tyro3 and Pros1. (TIF) [file pone.0208550.s002.tif]
