# Supplementary material for: Gas6 is dispensable for pubertal mammary gland development
Source: PLoS One. 2018 Dec 11;13(12):e0208550. doi: 10.1371/journal.pone.0208550 (PMC6289431; doi:10.1371/journal.pone.0208550)
Supplement: S1 Table — (DOCX) [file pone.0208550.s004.docx]

# Supporting information

# S1 table: List of antibodies

| Antibody | Application | Manufacturer | Catalog # | Dilution | Clonality | Host Species |  |
| --- | --- | --- | --- | --- | --- | --- | --- |
| Axl | WB | Santa Cruz | sc1097 | 1:1000 | Polyclonal | Goat |  |
| GAPDH | WB | Proteintech | 10494-1-AP | 1:10000 | Polyclonal | Rabbit |  |
| CK8 | IF | DSHB | TROMA-1 | 1:250 | Monoclonal | Rat |  |
| CK14 | IF | Biolegend | PRB-155P | 1:400 | Polyclonal | Rabbit |  |
| F4/80 | IHC | AbD Serotec | MCA497RT | 1:100 | Monoclonal | Rat |  |
| Gas6 | IHC | R&D | AF986 | 1:200 | Polyclonal | Goat |  |
| Ki67 | IF | Abcam | ab16667 | 1:50 | Monoclonal | Rabbit |  |
| Mer | WB | R&D | AF591 | 1:500 | Polyclonal | Goat |  |
| PanCK | IF | Abcam | ab86734 | 1:50 | Monoclonal | Mouse |  |
| Pros1 | WB | R&D | MAB4976 | 1:250 | Monoclonal | Rat |  |
| Tyro3 | WB | R&D | AF759 | 1:2000 | Polyclonal | Goat |  |
| CD24-BV711 | FC | BD Biosciences | 563450 | 1:100 | Monoclonal | Rat |  |
| CD29-e450 | FC | eBioscience | 48-0291-82 | 1:100 | Monoclonal | Armenian Hamster |  |
| CD14-FITC | FC | eBioscience | 11-0141-82 | 1:100 | Monoclonal | Rat |  |
| cKit-PE | FC | Cedarlane | CL8936PE | 1:50 | Monoclonal | Rat |  |
|  |  |  |  |  |  |  |  |
